# A dozen years of clinical trials performing advanced cell therapy with perinatal cells

**DOI:** 10.4155/fsoa-2018-0085

**Published:** 2018-10-26

**Authors:** Frances Verter, Pedro Silva Couto, Alexey Bersenev

**Affiliations:** 1 Parent's Guide to Cord Blood Foundation, Brookeville, MD 20833, USA; 2 Cell Therapy Laboratory at Yale-New Haven Hospital, Yale University, New Haven, CT 06510, USA

**Keywords:** cell therapy, clinical trial, cord blood, perinatal cells

Since we published our article [1], ‘The first decade of advanced cell therapy clinical trials using perinatal cells (2005–2015)’, we have continued to gather data on cell and gene therapy clinical trials, and we can now report on trends in perinatal cell therapy for the years 2005–2017.

**Figure 1.**
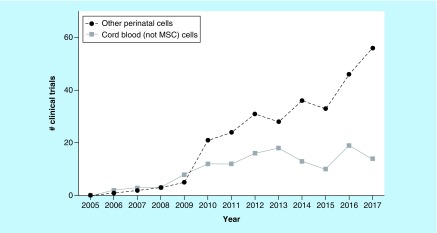
**Trends in the field of cell and gene therapy with perinatal cells.** MSC: Mesenchymal stem cells.

Figure 1 illustrates the most striking trends in the field of cell and gene therapy with perinatal cells. The number of clinical trials registered worldwide each year is flat for cells that are unique to cord blood, but rapidly growing for other perinatal cells. Since 2010, the number of clinical trials per year that rely on the action of perinatal cells unique to cord blood has been between 10 and 20 trials per year, averaging 14 trials per year. Note that this does not include standard hematopoietic stem cell transplantation trials because they do not meet our criteria of only archiving advanced cell therapy [2]. Meanwhile, the number of clinical trials that rely on cells from other perinatal sources doubled over the 5 years from 2013 to 2017, from 28 to 56. The majority of this growth, averaging 84% of the other perinatal trials since 2010, is from clinical trials that rely solely on mesenchymal stem/stromal cells (MSCs) from any perinatal sources, including cord blood, cord tissue and the placenta.

The rapid rise in clinical trials with MSCs from perinatal sources is even more striking when compared with the overall growth of advanced cell therapy with MSCs from any source. Our data at CellTrials.org show [3] that, over the 5 years from 2013 to 2017, the number of clinical trials that relied on the action of isolated MSCs from any source increased by 50%, whereas the number of trials with perinatal MSCs doubled during this time. As a result, the fraction of the MSC trials from perinatal sources rose from about 20 to 30%. In 2016 and 2017, trials with perinatal MSCs outnumbered trials with MSCs from bone marrow or adipose tissue.

Another clear difference between the advanced cell therapy trials that rely on cord blood cells versus other perinatal cells, is that perinatal MSCs have been applied to a much wider variety of indications for use. Admittedly diagnosis categories can be somewhat subjective, so we will clarify below which indications for use were included in each category.

Cumulatively from 2005 through 2017 there are a total of 131 advanced cell therapy clinical trials with cells unique to cord blood. Among these trials, 75% of the indications for use are either hematology/oncology (39%), or neurology (36%). No other diagnosis category has exceeded a 9% share of the trials with these cells over a dozen years. Since traditional stem cell transplants do not qualify as ‘advanced cell therapy’, the hematology/oncology trials we are counting here are focused on isolating and expanding specific cell lines from cord blood in order to enhance engraftment during transplants for malignancies or blood disorders. By comparison, most of the neurology trials are using unmanipulated cord blood for nonhomologous applications. The neurology indications include Alzheimer's disease, amyotrophic lateral sclerosis, autism, cerebral palsy, hypoxic-ischemic encephalopathy, Parkinson's disease, spinal cord injury and stroke.

Cumulatively from 2005 through 2017 there are a total of 238 advanced cell therapy clinical trials with perinatal MSCs. Among these trials, 64% of the indications for use fall into the five categories of autoimmune disorders (21%), and neurological (15%), cardiovascular (10%), orthopedic (9%) and liver diseases (9%). It should be noted that autoimmune disorders include conditions that have disparate symptoms but share auto-immune etiology, such as Crohn's disease, multiple sclerosis, psoriasis, rheumatoid arthritis, systemic lupus erythematosus and ulcerative colitis.

Thus, in the cord blood field advanced cell therapy clinical trials have been focused almost entirely on addressing two specific issues: engraftment during transplant and repair of acquired or degenerative neurologic damage. In contrast, perinatal MSCs have been trialed for almost any condition that might benefit from the immunomodulatory and anti-inflammatory properties of MSCs. This leads to the question of which trials, if any, have reached late phase and might culminate in an approved cell therapy product.

Through the end of 2017, 25 clinical trials of perinatal advanced cell therapies have reached Phase III or higher. Three companies have been responsible for all of the postmarket follow-up trials: Medipost, Gamida Cell and Osiris. Since each of these companies has a very different cell therapy manufacturing technology, we briefly review their product pipelines.

Medipost of South Korea has sponsored at least 17 advanced cell therapy clinical trials with perinatal cells so far, either alone or in collaborations, at locations in South Korea or the USA. In 2012 they achieved market approval in South Korea of their product Cartistem^®^ for degenerative arthritis, and they are currently seeking approval of products Pneumostem^®^ for bronchopulmonary dysplasia or hemorrhagic stroke, and Neurostem^®^-AD for Alzheimer's disease. The active cell type in all Medipost products is MSCs that are isolated from allogeneic cord blood and then expanded in culture.

Gamida Cell of Israel is seeking approval for their product, which is manufactured from allogeneic cord blood CD133^+^ cells that are cultured with the small molecule nicotinamide, and is called NiCord^®^ when used for hematologic malignancies or CordIn™ when the indication is hemoglobinopathies. They have sponsored at least ten trials at multinational locations and were the first stem cell transplant product to receive the US FDA Breakthrough Therapy Designation.

Osiris Therapeutics of the USA self-launched their Grafix^®^ wound dressing in 2011, and this product is included in follow-up studies conducted by wound registries. Grafix contains viable cryopreserved placental membranes; two versions of the product are Grafix Prime (amniotic membrane) and Grafix Core (chorionic membrane). Ironically, there are dozens more products manufactured from the amniotic membrane of the placenta that are marketed as wound dressings, but the majority of them do not contain live cells and therefore are not tracked by our database of advanced cell therapy clinical trials.

A few nations continue to lead the development of cell therapy products that rely on perinatal cells. We noted [1] that over the decade 2005–2015, only three countries accounted for 79% of the 281 advanced cell therapy trials with perinatal cells: China (36%), the USA (30%) and South Korea (12%). Since then, during the years 2016–2017, an additional 137 trials were registered to run at hospitals and clinics located in China (52%), the USA (18%), South Korea (7%) and elsewhere or multinational locations (23%). As before, nearly 80% of the trials are in the leading three nations, but recently over half the registered trials are in China.

At CellTrials.org [3] we noticed an odd coincidence: cumulative through the end of 2017, the worldwide number of advanced cell therapy trials with perinatal cells, which is 417, is very similar to the cumulative worldwide number of clinical trials with T-cells modified by Chimeric Antigen Receptors (CAR-T), which is 421. In recent years the success of CAR-T therapy has garnered enormous media attention. This illustrates that it only takes one dramatically successful study to propel a research area from relative obscurity into the limelight. We think that there is also a feedback pattern where regulatory approvals catalyze the growth of further clinical trials. Thus, the 2012 approval of Cartistem contributed to the growth of trials with perinatal MSCs, just as the 2017 approvals of CAR-T products Kymriah and Yescarta catalyzed that field. While CAR-T trials primarily target patients that have recently developed hematological malignancies, where the number of patients diagnosed per year is less than 0.2 million in the United States; by comparison, any one of the top five indications treated by trials of perinatal MSCs could be applied to millions of patients in the United States that have chronic conditions in those categories.

Based on the pipelines of clinical trials and regulatory approvals, we predict that more products manufactured from cord blood will achieve regulatory approval in the next couple of years. We also predict that the use of perinatal MSCs will continue to grow relative to MSCs from bone marrow or adipose tissue as a source of MSCs in clinical trials, both due to the relative ease of harvesting MSCs from perinatal sources as well as the greater proliferation ability of perinatal MSCs [4]. We predict that the use of placental membranes as a wound product will continue to grow, but most of these products will probably be decellularized and terminally sterilized, so that they are more accessible as an off-the-shelf therapy in the developing world.
